# Reconfiguring Plant Metabolism for Biodegradable Plastic Production

**DOI:** 10.34133/2020/9078303

**Published:** 2020-08-04

**Authors:** Haiwei Lu, Guoliang Yuan, Steven H. Strauss, Timothy J. Tschaplinski, Gerald A. Tuskan, Jin-Gui Chen, Xiaohan Yang

**Affiliations:** ^1^Biosciences Division, Oak Ridge National Laboratory, Oak Ridge, TN 37831, USA; ^2^The Center for Bioenergy Innovation, Oak Ridge National Laboratory, Oak Ridge, TN 37831, USA; ^3^Department of Forest Ecosystems and Society, Oregon State University, Corvallis, OR 97331, USA

## Abstract

For decades, plants have been the subject of genetic engineering to synthesize novel, value-added compounds. Polyhydroxyalkanoates (PHAs), a large class of biodegradable biopolymers naturally synthesized in eubacteria, are among the novel products that have been introduced to make use of plant acetyl-CoA metabolic pathways. It was hoped that renewable PHA production would help address environmental issues associated with the accumulation of nondegradable plastic wastes. However, after three decades of effort synthesizing PHAs, and in particular the simplest form polyhydroxybutyrate (PHB), and seeking to improve their production in plants, it has proven very difficult to reach a commercially profitable rate in a normally growing plant. This seems to be due to the growth defects associated with PHA production and accumulation in plant cells. Here, we review major breakthroughs that have been made in plant-based PHA synthesis using traditional genetic engineering approaches and discuss challenges that have been encountered. Then, from the point of view of plant synthetic biology, we provide perspectives on reprograming plant acetyl-CoA pathways for PHA production, with the goal of maximizing PHA yield while minimizing growth inhibition. Specifically, we suggest genetic elements that can be considered in genetic circuit design, approaches for nuclear genome and plastome modification, and the use of multiomics and mathematical modeling in understanding and restructuring plant metabolic pathways.

## 1. Introduction

As autotrophic organisms, plants have evolved sophisticated metabolic pathways to utilize sunlight and atmospheric carbon dioxide to produce a rich array of phytochemicals that are essential for plant growth and development. It has been estimated that there are 200,000 to 1 million distinct metabolites generated in plants [1]. The majority of the carbon fixed by plants, however, is lost due to respiration or is fixed in cell wall polymers [2]. To exploit plants for the production of customized compounds, considerable efforts have been made to engineer plant metabolic pathways [1–3]. In particular, biodegradable polyesters polyhydroxyalkanoates (PHAs), and especially its simplest form polyhydroxybutyrate (PHB), have been introduced as novel end products of acetyl-CoA anabolic metabolism. PHAs are being examined with the goals of mitigating the increasing dependence on plastic products in everyday life, the accumulation of a large body of petroleum-based nondegradable plastic wastes, and the consequent environmental and health issues [4–6]. To date, PHA yield accounting for up to 40% of dry weight (DW) has been demonstrated in *Arabidopsis thaliana* (Arabidopsis) [7]. Yet plant-based PHA production at a large scale remains challenging, largely due to the associated chlorosis and reduced growth observed in a number of cases [7–11].

In recent years, redirecting metabolic flux in microorganisms, including that for PHA synthesis, has been empowered by synthetic biology or “SynBio” [5, 12–14]. Inspired by integrated electric circuits that function in electronic devices, “SynBio” is aimed at building orthogonal biological parts into a genetic circuit that can predictably control the behavior of living organisms [15, 16]. “SynBio” relies on molecular technologies as much as traditional genetic engineering does. However, it distinguishes itself from classical genetic engineering in the emphasis on the ability to externally control gene expression and on the precision of gene expression sought in response to the external controls [16]. In addition, “SynBio” can be considered to be on a continuum with systems biology as it incorporates systems data and mathematical modeling to facilitate the understanding of the target organisms and to help ensure the resulting phenotype or behavior within set targets.

Compared with microorganisms, plants provide challenges in adapting some of the synthetic biology concepts. For example, quantitative prediction of behaviors of genetic parts (e.g., promoters, enhancers, and terminators), which are crucial for precise rewiring of metabolic pathways, is a daunting task, given the complex nature of land plants as multicellular organisms and the presence of multilevel regulation of gene expression [16]. Yet, proof-of-concept studies have proven the feasibility of identifying interchangeable genetic parts, delivering synthetic regulatory genetic circuits, and employing mathematical modeling in plant metabolic engineering [15, 17, 18]. With a focus on directing acetyl-CoA from endogenous metabolic pathways to PHA synthesis in plants, in this review, we provide our vision of how “SynBio” can be applied to the reconfiguration of plant metabolism for high levels of PHA production and minimal detrimental impacts on plant growth.

## 2. PHA Production in Plants: From Bud to Blossom

PHAs are a large group of polymers of 3-(R)-hydroxy fatty acids linked by an ester bond between the hydroxyl group and the carboxy group of an adjacent monomer. They are synthesized by most genera of eubacteria, typically under stress conditions to serve as carbon and energy storage compounds [5, 19]. As the simplest yet most representative form of PHAs, PHB has been actively studied since its initial discovery in the bacterium *Bacillus megaterium* in the early 1900s [20]. Biosynthesis of PHB requires acetyl-CoA as the substrate and three enzymes, *β*-ketothiolase (known as PhbA), acetoacetyl-CoA reductase (PhbB), and PHB synthase (PhbC), as catalysts (Figure 1(a), pathway I). Alternatively, with the presence of both acetyl-CoA and malonyl-CoA, acetoacetyl-CoA synthase (NphT7) from *Streptomyces* sp. can replace *β*-ketothiolase; it converts acetyl-CoA and malonyl-CoA to acetoacetyl-CoA for PHB synthesis (Figure 1(a), pathway II) [21]. The concept of synthesizing PHAs in plants at a cost that is comparable with petroleum-based plastics attracted the attention of the scientific community starting in about 1989, with the article “In search of the plastic potato” [22]. The required starting substrate for PHB synthesis—acetyl-CoA—is naturally produced in the plant cytosol and organelles, including plastids, mitochondria, peroxisomes, and the nucleus (Figure 1(b)). It serves as a key metabolite at the metabolic nexus—connecting catabolic and anabolic metabolism [23]. Compared with bacterial or yeast fermentation, crop plants, especially woody plants, are capable of producing large amounts of target compounds at large scale and low cost [19, 24]. It is estimated that a PHB production rate of 10-12.5% of DW in plants could be cost-competitive with petroleum-based plastics [17, 25–27].

Figure 1Biosynthesis of polyhydroxybutyrate (PHB) in bacteria and plants. (a) Bacterial pathways for PHB synthesis that have been engineered into plants. Pathway I (in orange) converts acetyl-CoA into PHB with three enzymes, PhbA (*β*-ketothiolase), PhbB (acetoacetyl-CoA reductase), and PhbC (PHB synthase). Pathway II (in blue) differs from pathway I in that it converts malonyl-CoA and acetyl-CoA to acetoacetyl-CoA with NphT7 (acetoacetyl-CoA synthase). Double arrows denote reversible reactions. (b) Visual summary of plant organelles that have been found to produce and/or accumulate PHB granules. Black arrows indicate endogenous acetyl-CoA metabolic pathways; orange and blue arrows indicate engineered PHB synthesis pathways using genes encoding enzymes in pathways I and II, respectively. Granules indicate the accumulation of PHB granules. PHB synthesis has been targeted in cytosol, plastids, and mitochondria, but no PHB has been produced in mitochondria. In addition, PHB has been observed in the nucleus and vacuoles in some studies, likely by transferring of the granules to these organelles. Figures and legend adapted from Oliver et al. [23] and Snell et al. [31].

**(a):**
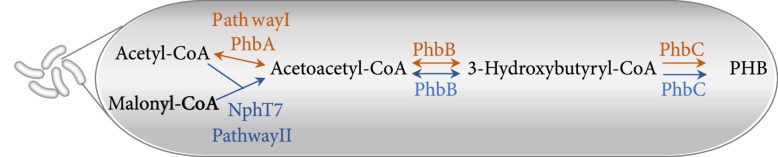

**(b):**
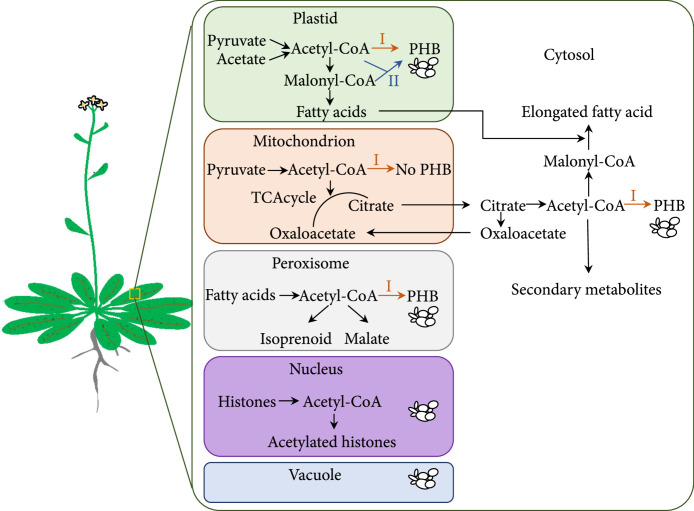

The first successful plant-based PHA synthesis was reported in 1992, which demonstrated that constitutive expression of the bacterium *Ralstonia eutropha* (formerly known as *Alcaligenes eutrophus*) genes, *PhbB* and *PhbC*, can lead to PHB granule accumulation in cytosol, nucleus, and vacuoles, in *Arabidopsis*, at a yield of 0.1% of DW [28]. The omission of the *PhbA* in the construct took advantage of the presence of endogenous *β*-ketothiolase in the cytosol. The accumulation of PHB in nucleus and vacuoles was likely due to the transfer of the granules from the cytosol to these organelles. This proof-of-concept study initiated a series of efforts to promote PHA production in plants [29–32]. In an incremental manner, expression of plastid-targeted *PhbA*, *PhbB*, and *PhbC* in *Arabidopsis* (by adding DNA fragments encoding a pea chloroplast transit peptide to the three PHB synthesis genes from *R*. *eutropha*) increased PHB production to 14% of DW [33]. In these early research efforts, each of the PHB synthesis genes was transformed individually into *Arabidopsis*, then combined via sexual crosses. Later, single constructs containing all of the genes for plastidial PHB synthesis were created, which further promoted PHB production, for example, to 40% of DW in *Arabidopsis* [7]. This is the highest PHB *in planta* yield that has been achieved to date. Meanwhile, PHAs have been successfully synthesized in peroxisomes, where acetyl-CoA is produced as the end product of fatty acid *β*-oxidation (Figure 1(b)). In *Arabidopsis*, peroxisomal PHA production has been achieved by expressing modified *PhaC1* from *Pseudomonas aeruginosa* [34, 35] or *PhbA*, *PhbB*, and *PhbC* from *R*. *eutropha* [36]. Besides the progresses made via nuclear genome modification, plastid transformation has been proven to be feasible for increasing PHB production, especially in tobacco [37–40]. By inserting native or modified forms of bacterial operons into the plastome (i.e., plastid genome), the PHB production rate has reached as high as 18.8% DW in tobacco [40], compared with a yield often below 0.3% of DW with nuclear transformation [41, 42].

In addition to model plant species *Arabidopsis* and tobacco, PHB and other forms of PHAs, including poly(3-hydroxybutyrate-co-3-hydroxyvalerate) (PHBV), short-chain-length (SCL) PHAs, medium-chain-length (MCL) PHAs, and SCL-MCL PHAs, have been synthesized in a number of crop plants which are more suitable for large-scale manufacture of these polymers [31, 32]. For example, industrial oilseed crops, including *Brassica napus* and *Camelina sativa*, have been examined as platforms for seed-based PHA production, with a yield up to 19.9% of DW [30, 43]. Sugarcane and switchgrass are two C_4_ biomass crops that have been most extensively tested for PHA production, particularly in the past decade [11, 17, 30, 31]. In sugarcane, the PHA synthesis pathways have been introduced to not only cytosol, plastids, and peroxisomes but also mitochondria [36, 44–48], where acetyl-CoA is used for energy generation (Figure 1(b)). However, no PHB accumulation was observed in mitochondria [44]. In C_4_ plants, it has been also found that PHA polymers preferentially accumulate in the plastids of bundle sheath cells with little to no polymers in mesophyll plastids [17, 31].

## 3. Factors Impeding Sufficient PHA Production

In the pioneering study reported in 1992, strong growth retardation and reduced seed production were found in PHB-producing *Arabidopsis* lines, despite low PHB accumulation at 0.1% of DW [28]. In this case, PHB was synthetized in the cytosol, where acetyl-CoA, the substrate for PHA production, is responsible for synthesis of other secondary metabolites (Figure 1(b)) that are essential for plant growth [23]. Because acetyl-CoA cannot be transported directly between the cytosol and other organelles, the diversion of cytosolic acetyl-CoA away from the endogenous metabolic pathways to PHA production has been proposed to be the main reason for disturbed plant growth and development [49]. In fact, the phenotypes of PHB-producing *Arabidopsis* plants have been found to resemble some of the phenotypes observed in plants with downregulated acetyl-CoA synthesis in the cytosol [50]. In agreement with these observations, overexpression of ATP citrate lyase (ACL), a cytosolic enzyme that generates acetyl-CoA and oxaloacetate from citrate and CoA, has been shown to be able to mitigate growth defects associated with cytosolic PHB synthesis in *Arabidopsis* [51].

Consequently, most of the subsequent efforts have been focused on synthesizing PHA in plastids, where higher abundance of acetyl-CoA is naturally available for fatty acid biosynthesis [33] (Figure 1(b)). Indeed, plastid-targeted synthesis has led to larger amount of PHA accumulation in general [30]. However, the presence of PHA granules in chloroplasts was also found to be problematic. For example, in the woody species poplar (*Populus*), negative effects on biomass growth and plant health were observed when PHB content exceeded 1% of DW [8, 19]. When produced in seed plastids of *Camelina sativa*, PHA accumulation can result in reduced seed oil content, germination rate, and seedling viability [43, 52, 53]. A series of C_4_ plant-based studies also found adverse phenotypes, such as stunting, chlorosis, and reduced biomass, especially with PHB yield above 1.5% of DW [9–11, 47]. It has been suggested that the accumulation of PHA granules in chloroplasts can shade thylakoids and disrupt the internal organization of chloroplasts, thereby leading to reduced photosynthetic efficiency and ATP starvation [11, 19]. To enhance photosynthesis, retransforming PHB-producing switchgrass with the bifunctional enzyme encoding gene *FBPase*/*SBPase* from *Synechococcus* was performed by Somleva et al. [30, 54]. The resulting PHA yield, although doubled what was observed in plants containing only the PHB genes, was only 7.7% DW, still lower than the commercially profitable yield (i.e., ≥10% of DW).

Another issue with C_4_ plant-based production is little accumulation of PHA in the plastids of mesophyll cells, which occupy 60%–70% of the total chlorenchyma area [31]. The uneven polymer accumulation was initially thought to be due to insufficient substrate availability or inefficient plastid targeting in mesophyll [44, 45, 55]. However, a nearly twofold increase in mesophyll production was accomplished in sugarcane and switchgrass by inhibiting the activity of acetyl-CoA carboxylase (ACCase), which catalyzes the first step of lipid biosynthesis and therefore competes with PhbA for acetyl-CoA [9, 48]. In addition, replacing PhbA with NphT7, which uses malonyl-CoA and acetyl-CoA for acetoacetyl-CoA production (Figure 1, pathway II), successfully promoted PHB synthesis in mesophyll and conferred up to 11.8% of DW PHB production in leaf samples of sugarcane [10]. Efficient plastid targeting in both bundle sheath and mesophyll cells with C_3_ dicot signals was proven to be successful by immunolocalization experiments [9]. These findings suggest that it is the availability and accessibility of acetyl-CoA in mesophyll cells, not the compatibility of the C_3_ dicot plastid targeting signal, that is the bottleneck to efficient PHB production in C_4_ plants.

**Figure 2:**
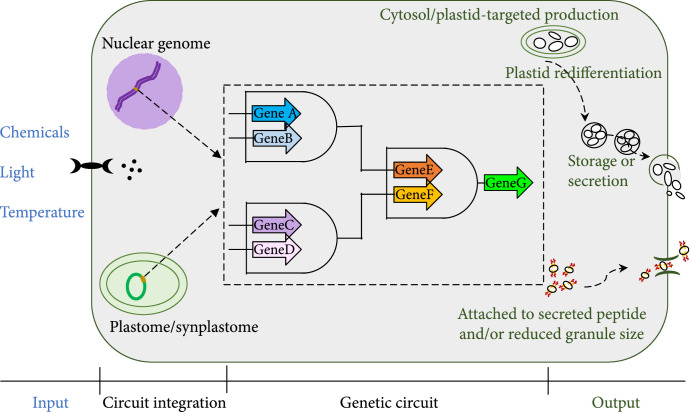
Elements to consider in genetic circuit design and genome modification for targeted synthesis, export, and storage.

**Figure 3:**
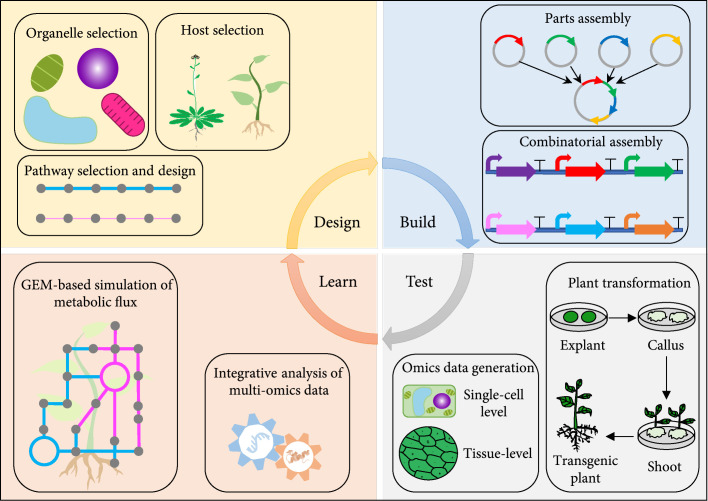
Informing PHA engineering with systems biology. Systems biology approaches (e.g., omics technologies, integrative analysis tools, and genome-scale metabolic models (GEMs)) can be used to inform the design-build-test-learn cycle of PHA engineering in plants by identifying biological parts for genetic circuit design and assessing the metabolic performance of PHA-synthesizing plants at systems level.

Guided by the groundbreaking studies mentioned above, in the sections below, we discuss several possible considerations for designing plant PHA synthesis constructs, delivering them into the plant genome (Figure 2), and utilizing systems data and metabolic modeling (Figure 3). We envision externally controllable PHA synthesis, by incorporating inducible or developmental stage-specific promoters into the genetic circuit with no or low impacts on plant growth and development. We also project targeted PHA synthesis, accumulation, relocation, and storage in dedicated organelles or attachments to secreted proteins.

## 4. Genetic Circuit for Controllable PHA Production and Storage

### 4.1. Precise Temporal Control of PHA Gene Expression

At present, growth defects associated with PHA synthesis seem to be inevitable. Future optimization efforts will require careful consideration of not only where in the plant the PHA is produced but also at what point in the plant’s lifecycle to avoid growth and development penalties. To date, several studies have suggested the superiority of inducible gene expression systems over constitutive gene expression systems in PHA biosynthesis. For example, replacing a constitutive promoter with a salicylic acid inducible promoter for plastid-targeted *PhbA* expression increased transformation efficiency in both tobacco and potato [42]. The use of an ethanol-inducible system for plastid-encoded PHB production solved previously observed growth deficiency and male sterility in tobacco plants engineered for constitutive PHB synthesis [38, 39]. In *Arabidopsis*, an ecdysone-inducible system led to improved health of PHB-producing plants in general [56]. In addition, a maize-derived light-inducible promoter was used for PHB synthesis in switchgrass [55]. However, these systems can be leaky [42, 56] and in some cases were not efficient in addressing the impaired plant health [42]. In addition, most of these studies reported a yield lower than the economically viable rate [39, 42, 55]. Despite that the ecdysone-inducible system was able to induce a yield up to 14% of DW in *Arabidopsis* [56], the system, when tested in poplar, only conferred a yield ranging 1-2% of DW [8].

Because there has been growing interest in synthesizing PHA in crop plants, and particularly in biomass crops, inducible systems suitable for use in field plantations are important. Among the inducible approaches discussed above, only the ecdysone-inducible system, which can be induced by certain types of commercially available pesticides [56], would attract broad interest. Clearly, additional systems need to be developed and tested. Developmental stage-specific promoters, such as senescence-associated promoters that have successfully altered biomass composition in *Arabidopsis* [57], hold promise for application in both greenhouse and field settings. However, if such approaches also reduce the development of cold and drought hardiness in perennial plants, this method might also have risks for plant health and productivity. Thus, it is essential to find the right promoters and to ensure their appropriate expression occurs (with regard to timing and intensity) even when inserted into a new genome position through transformation. High-throughput omics approaches now enable the identification of promoters at a large scale, providing many options for study. For example, in a poplar-based transcriptome study, a total of 68 sequence motifs were enriched among 1,712 genes found to be constantly upregulated during the onset and progression of seasonal leaf senescence [58]. These motifs hold considerable promise to be incorporated into inducible systems (i.e., genetic circuits) as genetic toggle switches to offer temporal control of PHA gene expression. Because leaf senescence is also regulated by multiple layers of epigenetic mechanisms [59, 60], one caveat in using senescence-specific promoters is that the effects of posttranscriptional modulation may give unexpected results. This includes mechanisms such as degradation by miRNAs and noncoding RNAs binding to *cis*-elements in mRNA [60]. Therefore, extensive fidelity testing in transgenic plants will be needed during promoter selection. It is possible to employ multiple tandem repeats in these promoters [61] to induce stronger senescence-specific PHA production. In addition, it may be possible to use multiple inducible or developmental stage-specific promoters to drive individual PHA synthesis genes in order to fine-tune the timing of gene expression. Finally, although plant insulator elements are not widely used, if effective they may be essential for providing reliable promoter expression, especially where the regulatory elements are nearby in constructs. A short transcriptional block from HIV [62] and/or the gypsy insulator with Hairy wing (Hw) binding protein from *Drosophila melanogaster* [63] should reduce promoter/enhancer interactions, though at the cost of increased construct size and complexity.

### 4.2. Increasing Production and Storage Capacity

When produced in plastids and other organelles, PHA accumulation is physically constrained by the size and the number of organelles within the cytoplasm and their storage capacity. Increasing the number and size of the organelles, therefore, is a logical path to promote PHA production. Plastids, like their free-living ancestors, cyanobacteria, proliferate through division of preexisting organelles. This process is orchestrated by ring-shaped contractile complexes with the coordination of nuclear gene encoding proteins, such as Plastid Division (PDV), Accumulation and Replication of Chloroplasts (ARC), Dynamin-Related Protein (DRP), and Min [64, 65]. In *Arabidopsis*, *arc* mutants have been found to have enlarged chloroplasts [66]. Suppression of the *phosphatidylinositol 4*-*phosphate* (*PI4P*) gene, which negatively regulates *PDV1* and *DRP5B* expression, can accelerate chloroplast division and increase the number of chloroplasts per cell [67]. These functionally characterized chloroplast division genes serve as promising targets for customizing the size and number of chloroplasts for enhanced PHA production and accumulation. However, manipulation of chloroplast division can result in reduced photosynthetic efficiency [66] and therefore compromised plant fitness and productivity. Also, there is likely a trade-off between the number and size of chloroplasts, because the total chloroplast compartment size is often closely related to the size and type of the cell [68, 69]. These factors may limit our ability to simultaneously increase chloroplast number and size in individual cells.

Because plastids have the ability to differentiate or redifferentiate in response to developmental and environmental cues [70, 71], it might be possible to control the redifferentiation of plastids from a PHA production organelle into a PHA transport or storage organelle. Chloroplasts have been found to be able to form vesicles that transport flavonoids to the vacuole [72]. In tomato fruits, chloroplasts can convert into chromoplasts—the pigment storage organelles—as carotenoid accumulates [73]. These naturally occurring processes may serve as valuable sources for understanding how chloroplasts form transport vesicles and redifferentiate into storage organelles.

### 4.3. Extracellular Storage via Secretion

In both bacteria and plant cells, PHA polymers form spherical granules after their synthesis. In bacteria, these granules are naturally attached to phospholipids and proteins, with PhaP1, the phasin protein, being dominant [74]. Bacteria devoid of phasins have been found to produce less PHB and display a slightly reduced growth rate. It has been thought that phasin production is a protective mechanism against the highly hydrophobic PHB granule [75]. In *Arabidopsis*, although expressing *PhaP* from *R*. *eutropha* in parallel with the PHB synthesis genes was not able to repair plant growth defects, phasins were detected on the surface of the PHB granules with a similar abundance to that in bacteria [42]. Taking advantage of the presence of phasins in *Escherichia coli* (*E*. *coli*), Rahman et al. [76] created a secretion system for phasin-bound PHB granules by fusing the *PhaP1* gene to the *HlyA* gene. Directed by the HlyA signal peptide, the resulting PhaP1 proteins and PHB granules were attached to these proteins and were able to be secreted via the type I secretion system. In addition, the presence of PhaP1 reduced the size of PHB granules, which facilitated the secretion of the granules together with the attached proteins. Although the secreted peptide-phasin-PHA fusion approach has not been tested in plants, similar fusion technology using oleosins, which are found in plants and have similar function to phasins, has been used in the production and isolation of hirudin—an anticoagulant for thrombosis treatment—in *Brassica* [77, 78]. Secreted peptides play essential roles in plant growth and development, and many of them, such as the Clavata3/Endosperm Surrounding Region- (ESR-) related root signal, are well characterized [79, 80]. By incorporating phasin protein and plant signal peptides into the PHA synthesis genetic circuit, it may be possible to relocate the PHA granules from within plant cells to extracellular spaces or even from production organs (e.g., leaves) to storage organs (e.g., roots).

## 5. Modification of the Nuclear and Plastid Genomes

### 5.1. Mitigating Chromosomal Context-Dependent Gene Expression

The integration of PHA synthesis constructs into the plant nuclear genome has relied on *Agrobacterium tumefaciens*-mediated transformation, which leads to random insertion of the PHA synthesis genes in the genome. Depending on the chromosomal context, the specificity of promoters, and consequently the expression of the transgenes, will vary widely. This context-dependent behavior presents a problem for PHA synthesis, which likely requires precise control, as discussed above. If endogenous promoters are used for driving PHA gene expression, inserting these genes into the plant genome precisely, as extension of native coding regions—for example, as protein fusions or inserting T2A or similar cleavable peptide linkers behind native protein coding regions [81]—may enable normal context-dependent expression. Site-directed insertion, though still challenging in plants, is feasible via genome editing approaches. For example, homologous recombination mediated by zinc-finger nuclease (ZFN), transcription activator-like effector nucleases (TALEN), and clustered regularly interspaced short palindromic repeats (CRISPR)/CRISPR-associated protein 9 (Cas9) has been used to successfully deliver herbicide-resistant genes into tobacco [82, 83] and maize [84] by modifying the acetolactate synthase genes—endogenes already present in the plant genomes.

CRISPR/Cas9 has become the most universal and user-friendly tool for fine targeted genome editing and is applicable to almost all living species. Most commonly, CRISPR/Cas9 systems are used to create loss of function via knockout of protein coding genes (e.g., small insertion/deletion mutations in the nucleic acid sequence and consequently creation of early stop codons and frame shifts that severely disrupt the amino acids in the protein product). However, many uses of gene targeting demand the replacement of some alleles with others or the insertion of genes into particular genome regions. Therefore, technology that can create gain of function mutations is in high demand. To date, the CRISPR/Cas9-mediated gene knock-in allows for one-to-one substitution of DNA sequence or the insertion of a gene in a specific locus in the plant genome. For example, a single-stranded *oligo* with a *Kpn*I+*Eco*RI site was introduced into the rice (*Oryza sativa*) phytoene desaturase gene *OsPDS* using protoplast transfection [85]. Also, gene replacement by homology-directed repair was accomplished with the presence of a DNA donor upon guide RNA (gRNA) combined with Cas9-mediated generation of a double-strand break in tobacco protoplasts [86]. However, in general, successful gene knock-ins in plants have been limited. One promising development, which generated in-frame gene knock-ins and amino acid substitutions, was accomplished by sequential transformation of *Arabidopsis* with a construct that induced germline-specific Cas9 expression together with gRNA and a donor sequence [87]. Other new precision gene editing tools include CRISPR-based prime editing which can perform targeted small insertions, deletions, and base swapping in a precise way without donor DNA [88]. Although prime editing was originally developed in yeast and human cells, the technique has been successfully applied to several plant species, such as rice and wheat [89–91]. Another new method is RNA-guided DNA insertion with CRISPR-associated transposases. Here, the reprogrammed transposases efficiently and specifically insert DNA both in vitro and into the *Escherichia coli* genome [92]. Similarly, another group reported a tool termed “insertion of transposable elements by gRNA-assisted targeting” that enabled highly specific, genome-wide DNA insertion across dozens of unique target sites in the *E*. *coli* genome [93]. Such CRISPR-mediated site-specific insertion, if applicable to plants and PHA biosynthesis regulons, may help mitigate context-variable behavior of promoters.

### 5.2. Plastome Modification

As an alternative to nuclear genome modification, plastome engineering provides a means to increase the expression level of PHA genes and consequently the yield of PHAs due to the polyploid nature of plastids. It has been estimated that there are approximately 500 to 5,000 copies of the plastid genome in a single plant cell [71]. Indeed, studies have confirmed that chloroplast transformation can lead to a very high level of transgene expression, which can exceed 70% of total soluble protein [94, 95]. Yet, reaching a profitable PHA yield via plastome modification is not as straightforward as expected. An early attempt, where the native *R*. *eutropha phb* operon was inserted into the tobacco plastome, showed only trace amounts (i.e., <0.05% of DW) of PHB accumulation [37], likely due to low genetic compatibility of the transgene cassette and poor transgene expression. Later efforts with optimized transgene expression cassette have been able to boost PHB yield. For example, the addition of a plastid-derived ribosome-binding site to each of the PHB synthesis genes in the native *R*. *eutropha* operon led to an increase in PHB yield to 0.016% of DW [96]. The fusion of a plastid promoter and plastid 5${}^{\prime }$ UTR to the native bacterial operon was able to raise PHB yield to 1.7% of DW during early tissue culture stages [38]. Subsequent greenhouse studies, however, showed a much lower yield of approximately 0.1% of DW. In addition, the PHB-producing tobacco plants exhibited growth deficiency and male sterility. These growth defects could be circumvented with inducible synthesis at late tissue culture stages [39].

The highest level of PHB production achieved with plastome modification so far—18.8% of DW in leaves (and 8.8% of DW of the whole plant)—was reported by Bohmert-Tatarev et al. [40]. The authors created a synthetic operon with the PhbA and PhbC encoding genes from *Acinetobacter* sp., the PhbB encoding gene from *Bacillus megaterium*, an antibiotic selectable marker, and multiple regulatory elements known to yield high levels of plastidial recombinant protein production. The three PHB synthesis genes were chosen because they had a similar codon usage and GC content to the tobacco plastome. Meanwhile, regulatory elements with limited homology to the host plastome were used to reduce unwanted rearrangements between regions of the transgenic insert and the host plastome. Although the PHB-producing plants tended to be paler green and smaller than wild-type tobacco plants, they were fertile and produced viable seeds. These studies indicate that with proper optimizations, the plastome engineering-based system is capable of promoting PHB yield by nearly 60-fold compared with nucleus-encoded plastid targeting strategy in tobacco [42]. With a number of plant species, such as cotton, soybean, sugarcane, oilseed rape, and poplar that are now amenable to plastid transformation [71, 97], plastome-targeted transgene cassettes could have a wider application in enhancing plant-based PHB production.

One inherent problem with plastid transformation, however, is low transgene expression in nongreen plastids. This phenomenon was reported in plastome engineering-enabled PHB synthesis (e.g., when the *phb* operon was inserted into the tobacco plastome as an extension of the *psbA* operon [40]), creating a bottleneck to efficient PHA production especially when seed plastids are being targeted. To exploit nongreen plastids for PHA production, regulatory sequences that can improve transgene expression in chromoplasts, amyloplasts, and other nongreen plastid types need to be incorporated into the plastid transgene. One candidate could be the plastid gene *clpP*. It encodes the proteolytic subunit of the Clp ATP-dependent protease and can induce a high level of plastid transgene expression in amyloplasts in potato [98] and root plastids [99].

Plastome engineering could also be combined with nuclear genome transformation to further boost PHA biosynthesis efficiency. This approach has been adopted in the inducible PHB synthesis system created by Lössl et al. [39], which consisted of a nuclear-located, ethanol-inducible T7RNA polymerase and a plastid harboring *phb* operon under the control of T7 regulatory elements. Yet another direction to pursue would be to introduce genes that encode enzymes promoting the acetyl-CoA flux in plastids. Fuentes et al. [100] employed a similar design to increase the yield of artemisinic acid, the precursor for the antimalaria drug artemisinin, in tobacco. The authors first inserted the core pathway for artemisinic acid biosynthesis into the chloroplast genome. Then, they introduced gene cassettes encoding enzymes that affect the flux of the artemisinin pathway into the nuclear genome. The retransformation with nucleus-targeted gene cassettes led to a maximum 77-fold increase in artemisinic acid production in the transplastomic lines without noticeable negative impacts on plant growth and development.

In addition to modifying the endogenous plastome, building an exogenous, truly synthetic plastome, termed “synplastome,” and introducing it into plastids are now feasible in algae and land plant species [101, 102], which opens up more possibilities for exploiting the biosynthetic versatility of plastids for PHA production and storage. If it is smaller and engineered only for promotion of PHA production, perhaps it would have less adverse effects than modifying natural chloroplast functions or numbers, as discussed above.

## 6. The Promise of Systems Biology for Promoting Plant-Based PHA Biosynthesis

### 6.1. Omics Empowered Identification of Genetic Parts

Systems biology, aiming for a holistic description and understanding of a biological system, encompasses a diversity of technologies and methods. As a major area of systems biology, omics reflect the totality of a specific type of molecular constituents (for example, DNA, mRNA, proteins, metabolites, and ions) within a biological organism. Omics data can inform “SynBio,” including PHA engineering, in several ways. For example, the knowledge gained through omics-based studies can help to enlarge the number of genetic parts which synthetic biologists could build upon (Figure 3). In plants, several types of omics data covering transcriptomics, epigenomics, proteomics, and metabolomics have been generated while studying leaf senescence [103]. Likewise, transcriptomics and proteomics have been used to study plastid differentiation [104]. The understanding of how biological components, such as individual genes, interact with each other during these developmental processes could support efforts in building genetic circuits for senescence stage-specific PHA synthesis or improved PHA production and storage capacity in plastids, as discussed in sections above.

Most omics data generated in plants, however, are based on a tissue- or organ-scale, therefore unable to distinguish cells that are physically close together yet process different developmental properties. For example, different developmental regulatory programs have been suggested to exist between plastids in epidermal, mesophyll, guard, and pavement cells, which can result in variations in the total number and size of chloroplasts in these cell types. In addition, divergence in thylakoid membrane development has been found among different cell layers of shoot apical meristem [70]. Uncovering mechanisms governing the variations among these physically connected cells requires cell type-specific omics technologies. These technologies employ tissue digestion and cell sorting during sample collection and are capable of capturing omics data from a specific type of cell or a single cell [105]. Although mostly applied in animals, single-cell omics can be adapted to plants to elucidate mechanisms of plastid differentiation, as evidenced by studies on root development in *Arabidopsis* [106] and alkaloid localization in *Catharanthus roseus* [107]. In addition, single-cell omics, when used to examine plant cells that produce PHAs, might provide insights into toxicity of these polymers.

### 6.2. Evaluating the Performance of PHA-Producing Plants with Integrative Analysis of Multiomics Data

Omics data and associated analysis tools provide means to analyze the functioning of PHA-engineered plants as systems and therefore troubleshoot performance issues (Figure 3), such as the perturbation of endogenous acetyl-CoA pathways and the adverse effects on plant growth and development. In fact, to explore limitations to PHB accumulation in sugarcane chloroplasts, McQualter et al. [11] examined and compared changes in mRNA, proteins, and metabolites in PHB-producing lines and wild-type plants. The findings suggested that the presence of PHB granules scatters photosynthetically active radiation and physically disrupts thylakoid membranes, both of which could lead to ATP starvation in bundle sheath chloroplasts.

Integrative analysis of multiomics data serves as an effective way to assimilate multiple datasets into biologically meaningful interpretations. This has been highlighted in comparative analyses that also examined different omics datasets independently, for example, that adopted by McQualter et al. [11] in analyzing PHB-synthesizing sugarcane. Despite the analytical challenges, omics integration methods have been successfully applied to several studies of plants [108]. For example, in analyzing oxidative stress responses in cambium in poplar, Srivastava et al. [109] integrated three datasets, including transcriptomics, proteomics, and metabolomics, using a modified orthogonal partial least squares multivariate regression method. In another poplar-based study that was focused on lignin biosynthesis, Wang et al. [110] developed an integration method consisting of transcript/protein equations, mass balance kinetic equations, and multiple linear regression equations. The authors applied the integration analysis to four datasets—transcriptomic, proteomic, fluxomic, and phenomic data generated from 221 transgenic poplar lines and 18 wild-type plants. This integrative analysis enabled the prediction of how changing the expression of any pathway gene or gene combination can affect protein abundance, metabolic flux, and phenotypic traits including lignin content and composition, tree growth, wood density, and wood saccharification potential. Clearly, there are opportunities for integrating multiomics data derived from PHA-engineered and wild-type plants, estimating perturbations as a result of PHA biosynthesis, and identifying directions for future optimization efforts.

### 6.3. Simulating Metabolic Flux in PHA-Producing Plants Using Genome-Scale Metabolic Models

As one of the major systems-based approaches for metabolic studies, in silico genome-scale metabolic model (GEM) construction had been considered beyond reach for plant species. Yet in the past decade, several GEMs have been constructed for *Arabidopsis*, rice, tomato, maize, sorghum, and sugarcane [111, 112]. Although different GEMs were developed with unique domain considerations (for example, for organelles that represent plant metabolic network and interactions that occur between bundle sheath and mesophyll), generally speaking, these GEMs computationally describe a whole set of gene-protein-reaction associations for the entire metabolic network in a given organism [112]. A key advantage of GEMs is therefore that they can be used to predict metabolic fluxes for an entire set of metabolic reactions. For example, an *Arabidopsis*-based GEM (named “AraGEM”) was used in a six-tissue context to explore C/N partitioning and resource allocation across leaf, stem, and root systems within a diurnal cycle [113]. Recently, a multitissue GEM (named “MultiGEM”) was applied to four spatiotemporal compartments, namely, bundle sheath, mesophyll, day, and night, with the goal of elucidating causes of growth retardation and low PHB production in mesophyll plastids in C_4_ plants [17]. The results revealed that several factors, including photoassimilation capacity, carbon availability, ATP maintenance, relative photosynthetic activity of bundle sheath and mesophyll, and type of metabolites exchanged in the plasmodesmata, can affect PHB yield in leaf samples of C_4_ plants. With multiple GEMs available for the model species *Arabidopsis* [112], similar pipelines can be used to identify bottlenecks to efficient PHA production, at least in this model species. For a broader application of GEMs, however, the gap between the number of plant species with established GEMs and the type of crop plants suitable for industrial-scale manufacture of PHA polymers needs to be filled. Nonetheless, the progresses in GEM construction and application have made it possible to generate a wide range of hypotheses to test in future PHA engineering studies (Figure 3).

## 7. Concluding Remarks

Using traditional genetic engineering approaches, many laboratories—working in a number of plant species—have reliably overexpressed the bacterial PHA pathways. However, the goal of reaching economically viable yields (e.g., a minimum of 10% of DW) without substantially disturbing plant development does not appear to be feasible by simple ectopic expression. Plant-based PHA synthesis likely requires careful consideration of the timing and duration of biosynthesis for organelle-targeted PHA production, possible relocation, and storage. The use of inducible or native developmental stage-specific promoters in newly constructed genetic circuits could potentially help reach this goal. These genetic circuits may also include genes required for maximizing the production and storage capability of plastids and other targeted organelles for production or facilitating the relocation and secretion of the PHA polymers (Figure 2). Meanwhile, the advancement of CRISPR/Cas9-mediated site-specific insertion technologies makes it feasible to insert PHA genes into the plant genome to construct native promoter “operons,” which might help mitigate the context-variable behavior of promoters and promote precise external control of gene expression. A main risk of this approach, however, is that it may be difficult to get the desired high level of PHA synthesis. Compared with nuclear genome modification, plastome engineering, or combining it with nuclear genome modification, is likely to confer higher expression of PHA genes, due to the polyploid nature of plastids. Alternatively, synplastomes engineered specifically for PHA production might have less adverse effects than modifying natural chloroplast functions or numbers. The possibilities for identifying additional genes and genetic circuit structures for optimizing PHA production have been greatly expanded by the incorporation of omics analytical tools and mathematical modeling (Figure 3). As we are entering the era of “SynBio,” it is a good time to reconsider bioplastics engineering in plants using a biosystems design approach.
